# Stereotactic Body Radiotherapy: Hitting Harder, Faster, and Smarter in High-Risk Prostate Cancer

**DOI:** 10.3389/fonc.2022.889132

**Published:** 2022-07-07

**Authors:** Rohann J. M. Correa, Andrew Loblaw

**Affiliations:** ^1^ Odette Cancer Centre, Sunnybrook Health Sciences Centre, Toronto, Canada; ^2^ Department of Radiation Oncology, University of Toronto, Toronto, Canada; ^3^ Division of Radiation Oncology, Department of Oncology, Western University and London Health Sciences Centre, London, Canada; ^4^ Department of Health Policy, Measurement and Evaluation, University of Toronto, Toronto, Canada

**Keywords:** high-risk prostate cancer, elective nodal irradiation (ENI), PSMA-PET, dose escalation, stereotactic body radiotherapy (SBRT)

## Abstract

Stereotactic body radiotherapy (SBRT) is a technologically sophisticated form of radiotherapy that holds significant potential to effectively treat high-risk prostate cancer (HRPC). Prostate SBRT has been the subject of intense investigation in the context of low- and intermediate-risk disease, but less so for HRPC. However, emerging data are demonstrating its potential to safely and efficiently delivery curative doses of radiotherapy, both to the prostate and elective lymph nodes. SBRT theoretically hits harder through radiobiological dose escalation facilitated by ultra-hypofractionation (UHRT), faster with only five treatment fractions, and smarter by using targeted, focal dose escalation to maximally ablate the dominant intraprostatic lesion (while maximally protecting normal tissues). To achieve this, advanced imaging modalities like magnetic resonance imaging and prostate specific membrane antigen positron emmission tomography (PSMA-PET) are leveraged in combination with cutting-edge radiotherapy planning and delivery technology. In this focused narrative review, we discuss key evidence and upcoming clinical trials evaluating SBRT for HRPC with a focus on dose escalation, elective nodal irradiation, and focal boost.

## Introduction

The National Comprehensive Cancer Network (NCCN) defines high-risk prostate cancer (HRPC) by any of the following high-risk features: ≥T3a, grade group ≥ 4, or PSA > 20. Very high-risk disease is a subset with any of the following: T3b-c, >4 cores of grade group 4 or 5, primary Gleason pattern 5, or any two high-risk features. Primary surgery can be utilized for HRPC but is associated with high rates of recurrence. In a study of 2,643 consecutive patients who underwent radical prostatectomy (RP) at a high-volume, tertiary care center, those with high-risk disease had a 5-year recurrence-free estimate of only 34.3% (1). Similarly, a European retrospective analysis of 4,041 men with high- and very high-risk disease who underwent RP demonstrated 8-year biochemical recurrence-free survival of 43.1% and 25.4% for the high- and very high-risk subsets, respectively (2).

Both radiotherapy and surgery are standard of care options for HRPC as per the NCCN (2022 update, version 3.0). These guidelines also permit the use of stereotactic body radiotherapy (SBRT) for high- or very high-risk disease, stating that “SBRT combined with ADT can be considered if delivering longer courses of EBRT would present a medical or social hardship.” Evidence continues to emerge in support of SBRT as a safe, tolerable, and effective option in HRPC. Herein, we review the literature supporting prostate SBRT for HRPC focusing on dose escalation and elective nodal irradiation (ENI) strategies, the impact of molecular imaging, and upcoming clinical trials.

## Dose Escalation for High-Risk Prostate Cancer

The value of dose escalation in HRPC is well established. Using brachytherapy (BT) boost, the landmark ASCENDE-RT trial set an important benchmark of long-term biochemical control achievable through whole-gland dose escalation (3). This randomized trial compared EBRT plus low-dose rate prostate BT (LDR-BT) boost versus dose-escalated (DE) EBRT, revealing significant improvement with BT boost: biochemical recurrence-free survival at 5 and 9 years were 86% and 83% versus 75% and 62%, respectively, and men receiving DE-EBRT were twice as likely to experience biochemical failure. However, these superior biochemical outcomes came at the cost of substantially increased toxicity in the BT arm: 5-year cumulative incidence of grade 3 genitourinary (GU) events were 18.4% versus 5.2% (4). High-dose rate (HDR) BT has also demonstrated superiority over conventionally fractionated radiotherapy (CFRT) for HRPC and is generally associated with less GU toxicity than LDR (5). Early randomized evidence from Sathya and colleagues demonstrated superior outcomes of HDR BT boost over CFRT, albeit to a relatively low dose of 66 Gy in 33 fractions by current standards (6, 7). Taken together, the biochemical outcome data following BT boost are a clear demonstration that HRPC requires escalation of dose beyond what is possible or practical with CFRT.

### Ultra-Hypofractionation and SBRT: Leveraging Radiobiology for Dose Escalation

Rather than dose escalating through ever-increasing fractions of CFRT, contemporary radiotherapy is instead moving toward fewer fractions and higher dose per fraction. Moderate hypofractionation (2.4–3.4 Gy per fraction)—and to a greater extent, UHRT (>5 Gy per fraction) (8)—aim to leverage the low α/β of prostate cancer to maximize biologically effective dose. The analysis by Vogelius and Bentzen using only randomised data (13 randomized controlled trials (RCTs), including hypofractionated and UHRT) has estimated the α/β for prostate cancers at 1.6 Gy (95% confidence interval (CI): 1.3–2.0) (9). Such models come with the caveat is that the linear quadratic model may not be accurate for larger fractional doses (over 6 Gy per day). Moreover, although this radiobiological rationale is intriguing and hypothesis-generating, empirical evidence is awaited to truly demonstrate the biological effectiveness of UHRT.

At present, the largest available randomized evidence for UHRT comes from HYPO-RT-PC, a non-inferiority RCT that randomized 1,200 prostate cancer patients to UHRT (42.7 Gy in 7 fractions) versus CFRT (78 Gy in 39 fractions)—including 126 high-risk patients. No androgen deprivation therapy was used. HYPO-RT-PC met its primary endpoint and demonstrated non-inferiority of UHRT, with 5-year failure-free survival (FFS) in both groups of 84% (95% CI: 80–87%) (10). Although equally effective, UHRT was associated with increased physician-reported GU toxicity (Radiation Therapy Oncology Group (RTOG) grade 2 or worse) at 1 year (*p* = 0.0037) (10). Patient-reported outcomes and quality-of-life (QoL) analysis correspondingly showed more GU/gastrointestinal (GI) symptoms acutely and more GU bother at 1 year in the UHRT arm. This greater toxicity might have been mitigated if true stereotactic technique was used. In HYPO-RT-PC, 80% of patients were treated with 7-mm planning target volume (PTV) margins and three-dimensional conformal radiotherapy. It is likely that contemporary planning and delivery techniques could achieve lower doses to organs at risk (OARs), which is known to correlate with toxicity and/or QoL (11).

SBRT can be considered a technologically advanced form of UHRT, leveraging technology for high-precision radiotherapy planning and delivery [e.g., image-guided radiotherapy (IGRT), modulated arc therapy (VMAT), robotic radiotherapy (CyberKnife), and magnetic resonance imaging (MRI)–guided or MRI-adaptive delivery]. In doing so, SBRT escalates dose while sparing OARs, thus maximizing the therapeutic ratio. True SBRT is being evaluated in the international PACE-B study, a non-inferiority RCT comparing SBRT to CFRT or moderately hypofractionated RT in 874 men. SBRT required implanted fiducials with 4- to 5-mm (3–5 mm posteriorly) PTV expansions. Prescription dose is specified as 36.25 Gy in 5 fractions to the PTV with a secondary dose target of 40 Gy to the CTV. IGRT delivery is mandatory (CyberKnife or conventional linear accelerator), and MRI-aided planning (fiducial-matched) is preferred. Although high-risk patients were not included in this trial, it is still relevant to consider the impact of rigorous SBRT technique on toxicity: PACE-B demonstrated that acute GU and GI toxicity rates were no different between SBRT and CFRT (12), in apparent contrast to HYPO-RT-PC. It should be acknowledged that the UHRT arm of HYPO-RT-PC used a higher PTV dose, which may also have contributed to higher toxicity. However, the rigorous standards for true SBRT technique in PACE-B, which were not mandated in HYPO-RT-PC, may also explain the isotoxicity of SBRT demonstrated in this trial.

Why was UHRT not superior to CFRT with respect to FFS in HYPO-RT-PC? After all, the equivalent doses in 2 Gy per fraction (EQD2) using an α/β of 1.6 Gy are 91.3 Gy versus 78 Gy for the UHRT versus CFRT arms, respectively. It is also interesting to note that HYPO-RT-PC was originally designed as a superiority trial (its sample size was increased from 800 to 1,200 at interim analysis to accommodate a revised, non-inferiority design). Explanations for isoeffectiveness may include statistical considerations, but a dosimetric explanation is also possible: because radiotherapy was “prescribed as mean PTV dose,” portions of the PTV received less/more than prescription dose by definition. Further dosimetric details were not specified (e.g., PTV/CTV coverage requirements, heterogeneity, and hotspots); therefore, it is conceivable that actual delivered dose to the CTV was lower than expected (which is known to correlate with biochemical recurrence-free survival in a dose–response relationship) (13). Conversely, it is also possible that in the absence of androgen deprivation therapy (ADT), there is a ceiling of effectiveness for local EBRT—even when dose-escalated *via* UHRT. However, without clarity on the proportion of local versus distant failure events, this is not certain. Ultimately, as we discuss below, further study of SBRT in the high-risk setting is needed, comparing against BT as the standard of care for dose escalation and with appropriate use of ADT.SBRT in HRPC: Emerging Evidence

Studies of SBRT including patients with HRPC have been reviewed exhaustively elsewhere (14), including a recent systematic review (15). However, the existing data are limited by several factors: predominantly retrospective studies, a wide range of SBRT techniques and dose/fractionation schedules utilized, short follow-up sometimes confounded by use of androgen deprivation therapy, and, chiefly, a relatively small proportion of study patients with high-risk disease who have been included.

An early pooled analysis from a multi-institutional consortium of prospective, phase II trials of prostate SBRT (King et al., 2013) included 125 patients with HRPC and demonstrated an encouraging a 5-year biochemical recurrence-free survival estimate of 81.2% (16). More recently, the SHARP consortium reported individual-patient data from patients with HRPC treated with SBRT (17). Their analysis included 344 patients (72% received ADT and 19% received ENI) who were followed for a median of 49.5 months. The estimated 4-year biochemical recurrence-free survival and distant metastasis-free survival rates were 81.7% and 89.1%, respectively. Interestingly, on multivariable analysis, lower dose (7 versus 8 Gy per fraction) was significantly associated with increased risk of biochemical failure (HR: 2.15; 95% CI: 1.07–4.32).

Here, we will focus on the few prospective studies that have investigated SBRT specifically for unfavorable or HRPC. Our discussion of these trials will encapsulate both lessons learned from early experiences and contemporary approaches in ongoing trials.

### Early SBRT Trials for HRPC: Millimeters Matter

Since 2001, Sunnybrook Hospital’s Odette Cancer Centre has explored increasingly hypo-fractionated, accelerated radiotherapy for prostate cancer through a series of iterative clinical trials, including for HRPC (11). In 2011, Sunnybrook launched one of the first prospective trials of prostate SBRT specifically for HRPC, enrolling 30 patients, 37% of whom had grade group 5 disease (18). Fiducial markers and daily IGRT were mandated, as was 12–18 months of ADT. Treatment volumes included the whole prostate (CTV2) plus proximal 1.5 cm of seminal vesicles (SV) or entire SV if T3b (CTV1). CTVs were expanded by 5 mm to create two separate PTVs. Radiotherapy dose was 40 and 30 Gy in 5 fractions to prostate and prostate + SVs, respectively. This SBRT regimen achieved a biochemical control rate of 85% and was generally well tolerated, with 0% and 3% late grade 3 GU and GI toxicity reported (11). However, the cumulative incidence of late hematochezia was notable at 42%. This was significantly higher than prior SBRT trials at Sunnybrook for low- and intermediate-risk disease that had treated prostate only (not including SVs). These trials utilized 35 Gy in 5 fractions (4-mm PTV margins) or 40 Gy in 5 fractions (5-mm PTV margin), yielding hematochezia rates of 4.9% and 27.2%, respectively (19). Combining these trial data, analysis of clinical and dosimetric predictors of hematochezia revealed that the volume of rectum receiving 38 Gy (V38) was a strong predictor of hematochezia. Furthermore, on multivariable analysis, V38 > 2cc, use of anticoagulants, and hemorrhoids emerged as the strongest predictive factors (19).

These important, empirical lessons regarding the safe delivery of prostate SBRT led to Sunnybrook’s next-generation SBRT protocol for HRPC: the SATURN trial (NCT01953055) (20). SATURN accrued 30 patients from 2013 to 2014 who were treated with 12–18 months of ADT and 5 fractions to the pelvis and entire SVs (CTV1) with a SIB to prostate alone (CTV2). PTV expansions of 6 and 3 mm generated PTV1 and PTV2, which were prescribed 25 and 40 Gy in 5 fractions, respectively. SATURN aimed to avoid the toxicity seen in prior studies by sharpening the penumbra and dose fall-off from the CTV. The prostate CTV received 99% of the 40-Gy prescription, whereas PTV1 and PTV2 received 23.75 Gy (95% of prescription) and 33.25 Gy, respectively. At a median of 72 months, there were zero biochemical failure events and no grade ≥ 3 GI or GU toxicities reported (21). Although the prevalence of grade 2 GU toxicity was 52% (persisting at last follow-up), it is important to note that 30% of patients had pre-existing grade 2 GU symptoms at baseline. Grade 2 GI toxicity prevalence was 24%, with 3% of patients reporting grade 2 GI symptoms at baseline (20).

Another early Canadian trial of SBRT was FASTR (NCT01439542) (22). This pilot study only enrolled patients with HRPC at the London Health Sciences Centre (London, Canada). Investigators targeted pelvic lymph nodes (CTV1) and prostate plus proximal 1 cm of SVs (CTV2). PTV expansions were 5 mm, and no fiducials or ancillary devices (e.g., rectal balloon) were used. PTV1 and PTV2 were prescribed 25 and 40 Gy (SIB) to 95% of their volume in 5 weekly fractions, respectively. IGRT with pre-fraction cone-beam CT (CBCT) was utilized. Unfortunately, FASTR was terminated early after the first 16 patients were accrued, owing to higher-than-expected rectal toxicity. Grade ≥ 3 GI events were seen in 25% (four patients), including 1 grade 4 event (bowel toxicity requiring partial colectomy). Analysis of dose–volume histogram parameters revealed that higher dose volumes to the rectum (i.e., volume receiving 20–40 Gy, or V20–V40) were most strongly associated with clinically significant bleeding. The FASTR investigators concluded that a larger high-dose CTV (including proximal SVs) and the 5-mm PTV margin likely account for the higher rectal toxicity observed (23). In essence, the prioritization of target coverage over OARs, among other factors, seemed to have contributed to the high rates of toxicity.

A subsequent trial, FASTR2, was launched from the same institution. This trial enrolled 30 patients with HRPC and very HRPC who were otherwise unable to complete a protracted course of CFRT due to frailty or geographic considerations. Unlike the original FASTR trial, in FASTR2, the posterior PTV margin was reduced to 4 mm, the dose was reduced to 35 Gy in 5 fractions, and tighter OAR constraints were used for rectum and bladder. This was better tolerated than the original FASTR trial, with no grade ≥3 toxicities and no rectal bleeding reported (24).

It is apparent from these pioneering SBRT trials in HRPC that small differences of a few millimeters in planning can make large differences when it comes to late rectal toxicity. Essential for safe delivery is IGRT with use of fiducials to tighten margins, as well as inverse radiotherapy planning (e.g., VMAT) to sharpen penumbra, achieve rapid dose fall-off, and respect normal tissue dose limits.

### SBRT With MRI-Enabled Focal Boost: Smarter Dose Escalation

Early SBRT dose-escalation studies have empirically established the limitations of dose to the whole gland. In low- and intermediate-risk disease, doses of 45–50 Gy in 5 fractions were associated with high toxicity, including grade IV cystitis and rectal complications requiring colostomy (25–27). Building upon these data and the pioneering HRPC SBRT studies discussed above, the next generation of SBRT trials seeks to achieve precision dose escalation of the dominant intraprostatic lesion (DIL), hitting HRPC harder in a smarter, more targeted fashion by leveraging advanced imaging technology.

The value of focal boost has now been established by the phase III FLAME RCT (28), which randomized 571 men with unfavorable disease (85% HRPC) to 77 Gy in 35 fractions (EQD2 of 81.8 Gy) to the whole prostate with or without focal simultaneous integrated boost (SIB) to 95 Gy (EQD2 115.8 Gy), targeting the MRI-defined DIL and reduced as needed to respect normal tissue constraints. At a median 72 months follow-up, the 5-year biochemical DFS was 92% with focal boost, significantly better than without (85%). Toxicity and QoL were favorable in both arms, and differences were small a not statistically significant. Likewise, a patterns of failure analysis of the FLAME trial demonstrated focal boost decreased both local and regional or distant metastatic failure (29). Although there was also a clear inverse relationship between achieved dose to the GTV and probability of biochemical failure, there appears to be saturation as the curve starts to plateau beyond 85–90 Gy (96–106 EQD2, α/β of 1.2) (28).

SBRT with integrated focal DIL boost is also being explored. One of the earlier studies evaluating this technique in HRPC was launched in 2017 at Sunnybrook and will soon read out its 5-year outcomes. The 5STAR trial (30) enrolled 30 patients with unfavorable disease (63% HRPC). All patients received prostate SBRT with focal DIL boost (35 Gy to prostate and up to 50 Gy to the DIL) plus ENI (25 Gy) in 5 weekly fractions. A fused MRI was used to delineate the DIL. A prostate PTV expansion of 2 mm (2.5 mm superior-inferiorly) was achieved with the use of fiducial markers and an endorectal immobilization device called GU-Lok (31). Nodal and prostate PTVs received 23.75 and 33.25 Gy, respectively. A CT urethrogram was done at time of CT simulation, and urethral Dmax was limited to <52 Gy. Daily CBCT was utilized for IGRT. The median DIL D90% delivered was 48.3 Gy (range: 45.2–51.9). No grade 3 events were observed. Cumulative grade 2 acute (<6 months) or late (6–24 months) GU toxicities were 67% and 46.7%, respectively, and GI toxicities were 16.7% and 13.3%, respectively. At approximately 5 years of follow-up, only a single patient (3.3%) has experienced biochemical failure in the form an out-of-field, non-regional nodal recurrence detected on PSMA-PET (abstract submitted to ASTRO 2022).

Other studies have also evaluated SBRT with focal boost in HRPC. The hypo-FLAME trial was launched to test DIL boost up to 50 Gy in the context of prostate SBRT (35 Gy in 5 fractions to the whole prostate). One-hundred men (75% HRPC) were enrolled and received a median D_mean_ of 44.6 Gy to the DIL. No acute grade ≥ 3 toxicity observed at a median follow-up of 18 months. Biochemical outcomes have not been reported at this early time point (32). The UK’s SPARC trial has also reported early data, specifically an interim safety analysis of eight patients with HRPC that received CyberKnife prostate SBRT (36.25 Gy to prostate) with up to 47.5 Gy boost to the DIL. Acute and late grade ≥2 toxicity was modest, the latter being 12.5% and 0% in the GU and GI domains, respectively. There were no biochemical failures in these eight patients after a median of 56 months follow-up (33).

Hannan and colleagues (34) recently reported a phase I trial of dose escalation to the DIL *beyond* 50 Gy. Fifty-five men with HRPC received pelvic and prostate SBRT with the prostate PTV prescribed 47.5 Gy and the DIL to 55 Gy in sequential cohorts. The pelvis received 22.5–25 Gy. Fused diagnostic mpMRI was used to delineate the DIL. Fiducial markers, hydrogel spacer, prophylactic alpha-blockers, and pre-fraction dexamethasone (4 mg) were utilized. A 2-year ADT course is planned. At a median follow-up of 18 months, grade 2 GI and GU toxicity was modest. One patient (lowest dose cohort) suffered a late grade 3 urinary retention requiring transurethral resection of prostate (TURP). A single biochemical failure was reported at 18 months, with subsequent development of widely metastatic disease in that patient. Although the technical achievement of focal dose escalation to this extreme is commendable, it is unclear whether the risk of increased toxicity is justified from an oncological perspective. The recently published FLAME trial patterns-of-failure analysis suggests that there are diminishing returns to dose escalation to the DIL in excess of 100 Gy EQD2 (α/β of 1.2), both in terms of local failure and regional/distant metastatic failure (29).

Molecular imaging with PSMA-PET imaging may also play an important role in focal DIL boost, in addition to its added diagnostic value (35) and superior accuracy for staging HRPC (36). Alfano and colleagues utilized PSMA-PET/MRI images co-registered with prostatectomy whole-mount histologic sections to determine a standard uptake value (SUV) threshold-based margin to aid in the accurate delineation of DILs for focal boost (37). Thus, it may be beneficial to fuse and integrate PSMA-PET imaging data into the contouring and planning workflow for DIL boost, and further study of this approach is warranted. Another challenge is so called “mpRMI invisibility” of some clinically significant lesions (38) and the known multi-focality of many prostate cancers (39). To address these challenges, the SPIRIT study is combining mpMRI, PSMA-PET, and whole-mount histology with genomic and methylomic analysis of malignant intraprostatic lesions (40). This has revealed a novel correlation between certain mpMRI higher-order radiomic features and genomic copy-number alteration, the latter of which is a surrogate for genomic instability and aggressive disease. In an expanded cohort, the next phase of this study aims to identify additional PSMA-PET/MRI radio-biologic correlations. In turn, these radiomic features could serve as “imaging biomarkers” to improve identification and delineation of the most aggressive lesions to facilitate focal boost.Elective Nodal Irradiation

The use of pelvic elective nodal radiotherapy (ENI) in HRPC remains controversial, chiefly due to a lack of overall or progression-free survival benefit in RCTs of ENI versus prostate-alone radiotherapy. This may be explained by several factors, and a nuanced discussion on this topic is beyond the scope of this focused review; moreover, it has been expertly reviewed recently (41). Definitive evidence is anticipated from RTOG 0924, which is assessing ADT and high-dose external beam radiotherapy to the prostate with or without the addition of whole-pelvis RT (NCT01368588). However, early results are expected to read out only after 2030. In the HRPC setting, trials evaluating increasingly hypofractionated ENI (combined with prostate boost) have demonstrated safety and favorable oncological outcomes, supporting the value of ENI in well-selected patients.

### Moderate Hypofractionation for ENI

In the high-risk setting, moderately hypofractionated prostate and pelvic EBRT has shown favorable outcomes. Once again, Sunnybrook was among the first to explore this approach through a study launched in 2004. This prospective, single-arm trial enrolled 230 patients with HRPC (prostate SIB to 68 Gy with 45 Gy in 25 fractions to the whole pelvis). The 5-year biochemical failure, distant metastasis, and overall survival rates were 15%, 6.6%, and 92.9%, respectively. Cumulative incidence rates of late grade ≥ 3 GI and GU toxicity were low at 2.5% and 7.5%, respectively. At 10 years, acceptable rates of biochemical failure, distant metastasis, and overall survival were maintained at 33.4%, 16.5%, and 76.3%, respectively (42).

More recently, the landmark trial POP-RT RCT randomized 224 men with HR and very HRPC to prostate only (68 Gy in 25 fractions) versus whole-pelvis RT (68 Gy prostate SIB with 50 Gy in 25 fractions to the pelvis, including common iliac nodes). At 68 months of median follow-up, biochemical FFS (bFFS) was remarkably high at 95% for whole-pelvis RT and significantly better than for prostate-only RT (81.2%). Disease-free survival and distant metastasis-free survival were also significantly higher for whole-pelvis RT, without a significant overall survival benefit (43). Of note, cumulative late GU toxicity (Gr. ≥ 2 RTOG) was significantly higher for whole-pelvis RT (20.0% *vs*. 8.9%), but neither late GI toxicity nor acute GU/GI toxicity differed between arms. The high rates of bFFS in POP-RT are likely attributable to several factors, including a higher ENI dose (50 Gy in POP-RT) and inclusion of common iliac nodes in the pelvic treatment volume. In addition, contributory was careful patient selection with inclusion of patients with HRPC at very high risk of nodal disease (median 38% by Roach formula) combined with the use of PSMA-PET staging in 80% to exclude occult nodal disease.

There is a great deal of enthusiasm around PSMA in HR PCa. The recent proPSMA trial supports the superiority of PSMA over conventional imaging with CT and bone scan for staging of HRPC (36). As PSMA-PET is increasingly adopted into routine practice, including for staging of HRPC, it will likely induce a so-called “Will Rogers” phenomenon (44), whereby outcomes for HRPC will appear to improve due to exclusion of early N+ and oligo-M1 disease identified by PSMA-PET (otherwise occult on conventional imaging). Average outcomes for the N+/oligo-M1 population will also improve as a consequence, because patients with occult (but PET-detectable) regional or distant disease will now be counted amongst those harboring conventionally detected, higher-volume nodal or metastatic disease. PSMA-PET also holds great potential to aid in the definition ENI treatment volumes. PSMA-PET studies have shown that elective volumes should be extended to cover to include the common iliac nodes up to the aortic bifurcation, thus providing justification for current NRG consensus contouring guidelines (45).

### Prostate and Pelvis SBRT: Ultra-Hypofractionated ENI With Simultaneous Integrated Prostate Boost

Using contemporary techniques for radiotherapy planning and delivery, anultra-hypofractionated course of ENI is possible. This approach is faster, more cost-effective, and more convenient for patients (18). It has been evaluated in the context of both prostate BT boost and SBRT boost. A recent pooled analysis evaluating four prospective clinical trials testing this “pelvic SBRT” approach in both contexts demonstrated its safety and tolerability in patients with unfavorable-intermediate risk (UIR) and HRPC (46). In 165 patients, worst grade 2 GU and GI toxicity rates were 48% and 7.5%, respectively. There were no grade 4 events and 2.7% of patients experienced grade 3 GU toxicity (0% GI). At a median follow-up of 38 months, late GU and GI toxicity rates (cumulative incidence of worst toxicity) were 41.1% and 10.5%, respectively. Grade 3 GY toxicity was 1.5%. Moreover, the strategy was associated with favorable rates of biochemical recurrence-free survival: 98% at 3 years, and no patient had pelvic failures. Pelvic SBRT following BT boost is currently being evaluated in ongoing RCTs including the HOPE (NCT04197141) (47) and SHARP (NCT04861415) trials based at London Health Sciences Centre and Sunnybrook Hospital, respectively. These trials are randomizing patients to UHRT ENI (25 Gy in 5 fractions) versus CFRT (37.5–46 Gy in 15–25 fractions) after single-fraction 15-Gy HDR BT boost.

An alternate approach that circumvents a BT procedure is the use of SBRT prostate boost plus ENI (CFRT or UHRT). One way to do this is with CFRT ENI and sequential prostate SBRT boost wherein 45–50.4 Gy is delivered in 1.8- to 2-Gy daily fractions to the pelvis followed by 19–21 Gy in 2–3 fractions to the prostate. Studies using this approach have been recently reviewed (14), and it is the subject of an ongoing RCT called PBS (Prostate Boost irradiation with SBRT) randomizing 100 men to CFRT versus SBRT boost (NCT03380806).

Alternately, and arguably more efficient and cost-effective, is the use of UHRT ENI with a prostate SIB. This is essentially simultaneous “SBRT to prostate and pelvis” and is a novel strategy that has also been explored in HRPC. The SATURN trial from Sunnybrook (discussed above) was one of the first to evaluate this paradigm (20), treating 30 patients with HRPC with 25 Gy in 5 fractions to the pelvis and 40 Gy in 5 fraction to the prostate (SIB). When individual patient data from SATURN were compared with a parallel trial of prostate-only SBRT, it was found that the addition of ENI did not significantly increase toxicity (11). Interestingly, there was also a trend toward superior biochemical control with the additional of ENI. Although intriguing, this *post hoc* comparison between two small prospective trials is hypothesis-generating at best but, nonetheless, paves the way for future prospective comparisons. The multi-center 5STAR-PC trial (n = 75) builds upon the initial 5STAR study (n = 30) discussed above, permitting the use of a focal DIL boost up to 50 Gy in the context of pelvic SBRT. 5STAR-PC has completed accrual and has reported favorable early (median 25 month) toxicity and QoL data. Encouragingly, there have been no biochemical failures reported thus far (46).

Murthy and colleagues have also evaluated pelvic SBRT (48). Using a prospective registry, 68 patients with HR (30%), very HR (16%), and N+ (54%) patients were treated with SBRT to the prostate and entire SVs (35–37.5 Gy), pelvis (25 Gy), and gross nodes (35–37.5 Gy) in 5 fractions. PTV expansion was 5 mm for elective and gross nodes, whereas prostate and SVs were expanded 3 mm posteriorly. Patients were also treated with neoadjuvant, concurrent, and adjuvant ADT (median 15 months total). SBRT was well tolerated with no acute grade 3 events. Grade 2 GU and GI toxicity rates were 12% and 3%, respectively. Although median follow-up was only 18 months, late toxicity rates was also favorable, with two patients experiencing grade 3 GU toxicity. Grade 2 GU and GI event rates were also very low at 4.5% and 4%, respectively. Encouragingly, 94% of patients were biochemically controlled and none of the N+ patients recurred, albeit with very limited follow-up.

## Future Directions

**Figure 1:**
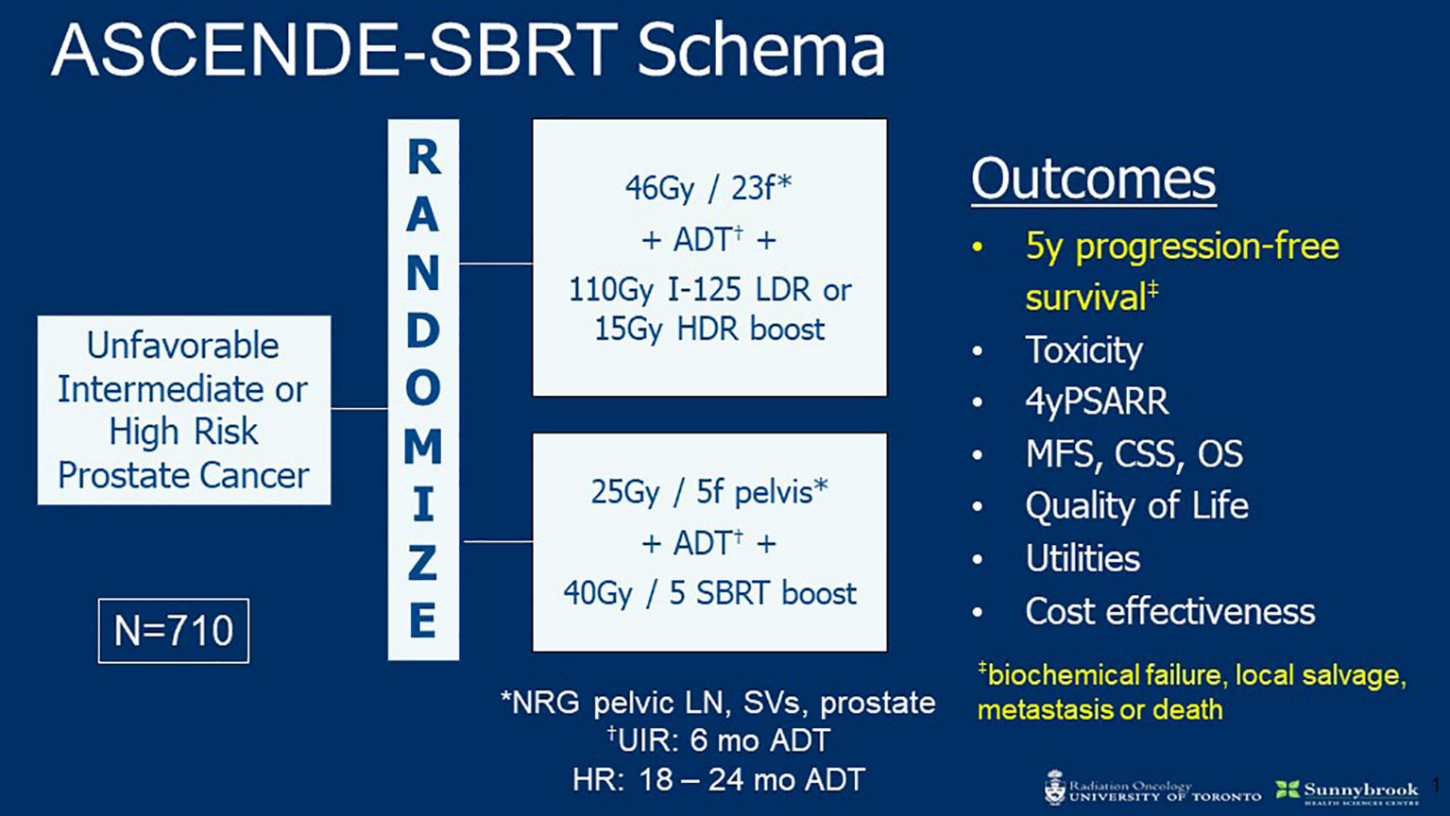
ASCENDE-SBRT Trial Schema.

The encouraging data reviewed above establish a strong rationale to evaluate prostate and pelvic SBRT in a randomized setting. The phase III ASCENDE-SBRT trial is launching soon to do so (**Figure 1**). This multi-center RCT will enroll 710 patients with UIR or HRPC and randomize to ENI plus BT versus SBRT boost. The BT arm will receive CFRT (46 Gy in 23 fractions) following HDR (15 Gy) or LDR (115 Gy I-125) BT boost. The SBRT arm will treat with 25 and 40 Gy in 5 fractions to the pelvis and prostate, respectively. Patients in both arms will receive ADT (4–6 months for UIR and 18–36 months for HR). The primary outcome is 5-year PFS (includes biochemical failure, local salvage, metastasis, or death) and is powered for non-inferiority. Secondary endpoints include toxicity, 4-year PSA response rate, metastasis free survival (MFS), cause specific survival (CSS), overall survival (OS), QoL, and cost effectiveness. The hypothesis of ADCENDE-SBRT is that SBRT prostate boost with simultaneous ENI will achieve similar outcomes to BT boost followed by ENI.

Other important trials are evaluating SBRT in HRPC. The PRIME trial (NCT03561961) is a phase III RCT comparing the efficacy of moderate hypofractionation (50 Gy to pelvis and 66–68 Gy to prostate in 25 fractions) with SBRT (25 Gy to pelvis, 35–36.5 Gy to prostate, and 30–35 Gy to gross nodes all in 5 fractions) in HRPC and N+ prostate cancer. The primary outcome is bFFS at 5 years, and it is powered for non-inferiority with a target accrual of 464 men. Another trial that will evaluate SBRT in HRPC—specifically pelvic SBRT—is PACE-NODES. Akin to POP-RT, which compared moderately hypofractionated RT to prostate only versus whole pelvis, this RCT will compare prostate-only versus prostate and pelvis SBRT, evaluating acute and late toxicity as well as bFFS in men with localized HRPC.

The increasing importance of PSMA-PET in the management of prostate cancer raises interesting questions regarding regional nodal disease. For instance, how should small PSMA-avid lymph nodes that do not meet size criteria by conventional imaging be managed? In the context of pelvic RT (including SBRT), what is the optimal nodal boost dose that adequately balances efficacy with potential toxicity? It will thus be important to conduct further study of PSMA-PET imaging as this technology integrates into the management pathway of HRPC. The ARGOS/CLIMBER trial, which is opening jointly through London Health Sciences Centre and Sunnybrook Hospital, is directly addressing this. It is discussed in a separate article by Liu and colleagues in this issue of *Frontiers Oncology*.

Future trials will also need to account for the rapidly evolving landscape of systemic therapy for advanced prostate cancer, and how this impacts the management of HR disease. The recently published STAMPEDE-platform RCT of abiraterone acetate (and prednisone) with or without enzalutamide for non-metastatic HRPC demonstrated significant oncological benefit with addition the former drug to ADT (49). It is interesting that protocol radiotherapy for this trial is described as “treatment of the prostate and SVs to 74 Gy in 37 fractions or equivalent hypofractionation” (i.e., does not appear to include elective nodes). Thus, in the context of systemic therapy intensification for HRPC, the role of ENI as well as whole-gland dose escalation (with BT or SBRT) and/or focal dose escalation with micro-boost would require further investigation.

## Conclusion

Contemporary radiotherapy has achieved excellent outcomes for patients with HRPC, as evidenced by the results of several prospective trials, including phase III studies such as FLAME and POP-RT discussed above. Such promising data have led some to wonder whether high-technology radiotherapy will emerge as the favored treatment option over surgery for HRPC (50). SBRT holds great potential to achieve favorable outcomes in HRPC. It encapsulates technologically driven UHRT for radiobiological dose escalation and can deliver simultaneous ENI and MRI-directed focal boost. SBRT has the potential to hit harder, faster, and smarter and all for less cost and greater convenience for patients. This paradigm is now being tested at the phase III level, with international RCTs like the ASCENDE-SBRT study launching soon.

## Author Contributions

RJMC and AL: writing and revising. All authors contributed to the article and approved the submitted version.

## Conflict of Interest

RJMC: None. AL: Grants/research support: TerSera and Tolmar; Honoraria/travel: AbbVie, Astellas, Bayer, Janssen, Knight, Merck, Sanofi, and TerSera; Advisory boards/consulting: AbbVie, Astellas, Janssen, Sanofi, TerSera, and Tolmar.

## Publisher’s Note

All claims expressed in this article are solely those of the authors and do not necessarily represent those of their affiliated organizations, or those of the publisher, the editors and the reviewers. Any product that may be evaluated in this article, or claim that may be made by its manufacturer, is not guaranteed or endorsed by the publisher.
